# Sinusoidal Microchannel with Descending Curves for Varicose Veins Implantation

**DOI:** 10.3390/mi9020059

**Published:** 2018-01-31

**Authors:** Muhammad Javaid Afzal, Muhammad Waseem Ashraf, Shahzadi Tayyaba, M. Khalid Hossain, Nitin Afzulpurkar

**Affiliations:** 1 Department of Physics, The University of Lahore, Lahore 54000, Pakistan; javaidphy@gmail.com; 2 Department of Physics (Electronics), GC University, Lahore 54000, Pakistan; 3 Department of Computer Engineering, The University of Lahore, Lahore 54000, Pakistan; shahzadi.tayyaba@hotmail.com; 4 Institute of Electronics, Atomic Energy Research Establishment, Bangladesh Atomic Energy Commission, Dhaka 1349, Bangladesh; khalid.baec@baec.gov.bd; 5 Department of Mechanical Engineering Technology (MCET), Higher Colleges of Technology (HCT), Ras al-Khaimah P.O. Box 4793, UAE; afzulpurkar.n@gmail.com

**Keywords:** descending sinusoidal microchannel (DSMC), varicose vein, fuzzy logic, ANSYS, biomedical microdevice, tissue damage

## Abstract

Approximately 26% of adult people, mostly females, are affected by varicose veins in old age. It is a common reason for distress, loss of efficiency, and worsening living conditions. Several traditional treatment techniques (sclerotherapy and foam sclerotherapy of large veins, laser surgeries and radiofrequency ablation, vein ligation and stripping, ambulatory phlebectomy, and endoscopic vein surgery) have failed to handle this disease effectively. Herein, authors have presented an alternative varicose vein implant method—the descending sinusoidal microchannel (DSMC). DSMC was simulated by Fuzzy logic MATLAB (The MathWorks, Natick, MA, USA) and ANSYS (ANSYS 18.2, perpetual license purchased by Ibadat Education Trust, The University of Lahore, Pakistan) with real and actual conditions. After simulations of DSMC, fabrication and testing were performed. The silver DSMC was manufactured by utilizing a micromachining procedure. The length, width, and depth of the silver substrate were 51 mm, 25 mm, and 1.1 mm, respectively. The measurements of the DSMC channel in the silver wafer substrate were 0.9 mm in width and 0.9 mm in depth. The three descending curves of the DSMC were 7 mm, 6 mm, and 5 mm in height. For pressure, actual conditions were carefully taken as 1.0 kPa to 1.5 kPa for varicose veins. For velocity, actual conditions were carefully taken as 0.02 m/s to 0.07 m/s for these veins. These are real and standard values used in simulations and experiments. At Reynolds number 323, the flow rate and velocity were determined as 1001.0 (0.1 nL/s), 11.4 cm/s and 1015.3 (0.1 nL/s), 12.19 cm/s by MATLAB (The MathWorks, Natick, MA, USA) and ANSYS simulations, respectively. The flow rate and velocity were determined to be 995.3 (0.1 nL/s) and 12.2 cm/s, respectively, at the same Reynolds number (323) in the experiment. Moreover, the Dean number was also calculated to observe Dean vortices. All simulated and experimental results were in close agreement. Consequently, DSMC can be implanted in varicose veins as a new treatment to preserve excellent blood flow in human legs from the original place to avoid tissue damage and other problems.

## 1. Introduction

**Figure 1:**
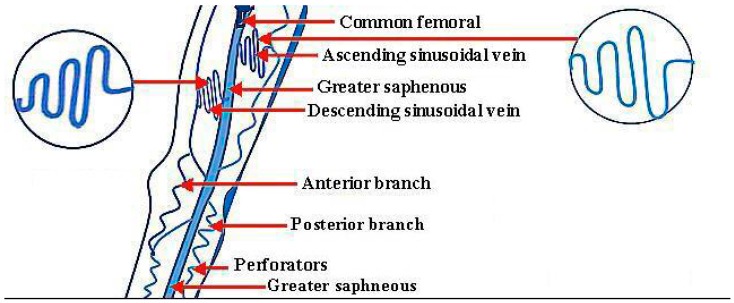
Normal veins which can become varicose veins.

A varicose vein is an enlarged and disrupted vein which is visible under the skin. They often seem swollen and blue in color, which are abnormal conditions. Major complications are found in the lower legs in humans. These varicose veins become worse with time. This crucial disease is actually an enlarged and stretched vascular form in the lower legs, and has been reported as a common chronic condition in medical science. The latest research has recognized a marital clustering of this disease. The genomic component is usually recognized, as well as the influences of age, profession, pregnancy, and obesity. The morphological features of this disease have been very well documented [1]. Tortuous veins can become varicose veins, and are seen with several shapes, lengths, and diameters [2]. These veins can have any shape. The ascending and descending sinusoidal veins are shown in Figure 1 below. 

Their lengths and diameters can be in the micrometer range, and even in the nanometer range. There are two aspects related to the types of varicose veins. One is the location of occurrence of varicose veins in the human body, and the second is the variety and intensity of appearance of varicose veins. In the human body, the first type of varicose vein is the great saphenous vein, which is linked to the femoral vein from the inner part of the ankle. The second type is the lesser saphenous vein, which is linked to the deep veins at the back side of knees. The third is the branch-type vein, which is linked with the side veins that are branched with the great saphenous vein. The fourth is the genital area vein, which is linked from the groin to the back side of the femur and near the womb and ovary. The fifth are the reticular and web-type veins, which are found anywhere on the human body. As for the variety in the intensity of appearance of these veins, they have four types. Two of them have severe and moderate intensity. The third is related to pregnancy, and the fourth type is known as the spider vein, and can be found anywhere [3,4,5,6,7,8,9].

The conventional treatment methods—self-care (exercise, weight loss) and compression stockings—can ease pain and prevent the disease from getting worse. The additional treatment methods for varicose vein disease are described below.

### 1.1. Sclerotherapy and Foam Sclerotherapy of Large Veins

In this invasive treatment method, a solution or foam solution is injected in varicose veins. After some days, the varicose veins should disappear. However, there is a risk involved with this type of treatment method, such as deep vein thrombosis (DVT) transmitting the risk of pulmonary embolism. Therefore, it is not a safe treatment method [2,10,11].

### 1.2. Laser Surgeries and Radiofrequency

The use of laser treatment to treat minor varicose veins is a new technology. This type of surgery works by transporting laser light spurts in to the veins, or a thin catheter can be inserted into an enlarged varicose vein and the high temperature of the catheter tip treats these veins with radiofrequency or laser. It is a non-invasive method, but there is a risk of hyperpigmentation [2,7,12].

### 1.3. Vein Ligation and Stripping

Vein litigation and stripping is an invasive method, and the removal of a main vein can cause tissue damage when the blood moves from small veins as the main vein is removed [2,13,14].

### 1.4. Ambulatory Phlebectomy

In this treatment method, there is a removal of smaller varicose veins with a long sequence of small punctures in the skin. It is very painful method with complications of imperfect removal bruising, intra-arterial injection, hyperpigmentation, skin necrosis, and swelling of varicose veins that can lead to other diseases [2,15,16].

### 1.5. Endoscopic Vein Surgery

Endoscopic vein surgery is a major operation in severe cases, and involves the removal of leg ulcers. In this method, a tiny video camera is injected into the legs and observed from inside. These veins are then removed through surgery. This treatment comes with a rapid onset of side effects after surgery which include pain, tissue damage, bruising, skin discoloration, and swelling. After-effects are very severe with ligation and stripping [2,12,17,18]. 

The main reason for the failure of all these treatment methods is the tissue damage. These traditional methods cannot stop tissue damage. Consequently, the idea of a very safe treatment method has been introduced, which is the implant of silver bioengineered vein as varicose vein. No tissue damage loss occurs with the use of this technique. The biocompatibility of the fabrication material of these microchannels has been discussed in our previous paper [2]. For this method, there is a need of straight, curved, spiral, circular, ascending, and descending sinusoidal microchannels which can act like bioengineered veins. The shape of varicose veins is exactly like these microchannels. This study focuses on the descending sinusoidal microchannel (DSMC). To date, microchannels have been used broadly in biomedical applications in the field of bioMEMS (bio-microelectromechanical systems) [19]. Huang et al. (2017) worked on bioMEMS device with horizontal straight microchannels for effectual 3D rotation and solitary cell loading [20]. Wang and Fan (2017) worked on stretching DNA with microchannels [21]. Faustino et al. (2016) worked on low-cost microfluidic devices with biomedical applications [22]. Hu and Ohta (2017) worked on cell manipulation in microdevices [23]. Birch and Landers (2017) worked on microfluidic systems for DNA handling and separation [24]. Pinto et al. (2017) worked on blood flow analysis with the help of biomedical microdevices [25]. Rodionov et al. (2013) worked on the brain implant of intracranial electroencephalogram electrodes [26]. Gibney Elizabeth (2015) reported on a brain implant for neurons [27]. Afzal et al. (2017) worked on an ascending sinusoidal microchannel (ASMC) for the treatment of varicose veins disease [2]. Sundell et al. (2017) worked on humanoid dental implants at the atomic scale [28]. Slepian et al. (2017) worked on a cardiovascular implant in the heart [29]. Dillon et al. (2017) worked on an intraocular lens with a revised technique for iris suture [30]. Abdollahi et al. (2017) worked on a cochlear implant for kids [31]. Ramesh et al. (2017) worked on metal stents for the heart [32]. Wang et al. (2017) worked on artificial dermis implantation for the replacement of dead skin [33]. Furthermore, the medical devices as an implant in human body are listed below in Table 1.

An ASMC was simulated, fabricated, and tested in the previous paper, with the idea of implantation in the human body. All the surgical problems for implantation were discussed in the first part of the study. ASMC cannot be used in every place because of velocity and flow rate difference [2]. Therefore, a new microdevice, DSMC, has been simulated using ANSYS (ANSYS 18.2, perpetual license purchased by Ibadat Education Trust, The University of Lahore, Pakistan) and MATLAB (The MathWorks, Natick, MA, USA), fabricated and tested for varicose veins near the thigh bone and in other places where DSMC is necessary to implant for increased blood flow rate and velocity.

## 2. Blood Rheology

**Figure 2:**
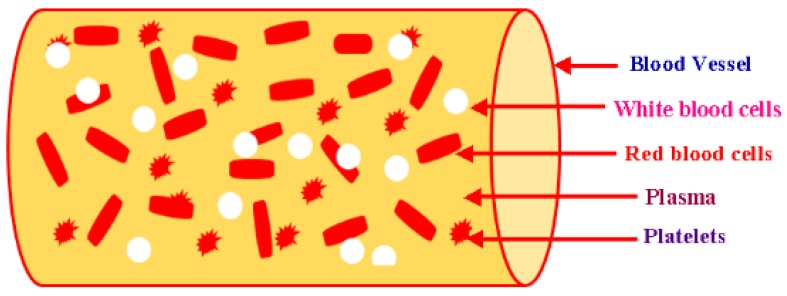
Blood and its components.

The human body is composed of 7% blood by weight. A mature person has 10 pints of blood in the body. The investigation of the flow characteristics of blood (plasma and cells) is called blood rheology. Suitable tissue perfusion occurs when the rheological blood characteristics are within certain ranges. Changes in the characteristics can play an important role in the growth rate of diseases. The viscosity of blood is obtained by plasma and the hematological values of red blood cells (RBCs) [35]. As a fluid, blood has non-Newtonian behavior, and the viscidity of blood changes intrinsically with the shear rate and temperature. The viscosity of blood has an inverse relationship with the temperature. When blood becomes less viscous, it develops high shear rates for systolic blood pressure. In the course of diastolic pressure, blood travels very slowly. Therefore, it develops thickness. Thus, blood is taken as a shear-thinning liquid [36]. Humanoid blood has a property named as viscoelasticity. The distortion of RBCs (red blood cells) is the main reason for the storage of elastic potential energy because of the pumping of heart. Blood constituents are shown below in Figure 2.

This elastic potential energy is transmitted to the blood by the pumping of the heart. It is stored to some extent in the elastic edifice of blood vessels, and the other smaller part is vanished due to viscosity. The residual energy is the reason for blood’s kinetic movement. Blood rheology is helpful in making the choice of the model being used in ANSYS Fluent. The density (1048 kg/m^3^) and viscosity (0.0032 kg·m^−1^·s^−1^) of blood was measured by rheometer for this research. Blood was taken as a non-Newtonian fluid in this study; the measured rheological data of the blood sample presents the non-Newtonian rheological property, which can help to determine the non-Newtonian viscosity model to be used in the ANSYS simulation. The optimal model can be determined by the consideration of the blood flow physics. In research, there is always the requirement of conventional practice for a definite problem and the level of accuracy. In simulation through ANSYS Fluent software, scientists always use ten different models, depending on the research problem. Among them, the K-epsilon model was selected due to the flow conditions in the set up tool of ANSYS. K-epsilon is a perfect model for the elucidation of two more equations: turbulent kinetic energy and its dissipation (both are zero in laminar flow). ANSYS simulation was done in order to provide the actual blood flow situation. In blood-bloated veins, velocity decreases due to friction from the blood hitting the walls of the vessels during its flow. In this case, the K-epsilon model was used as a reference setpoint. The K-epsilon model is a turbulence model. The turbulence models can present effects which are not physical in blood flow, even if the flow is purely laminar. Furthermore, scientists have nominated this model in several research works [2,37,38,39,40,41,42,43].

## 3. Fuzzy Logic MATLAB Simulation for Descending Sinusoidal Microchannel (DSMC)

Fuzzy logic controller (FLC) was managed to evaluate various parameters of DSMC. This system had the same input and output parameters with same membership functions. Their ranges were set according to the descending nature of the DSMC. In the Fuzzy logic MATLAB technique, there are three membership functions taken for each input and output variable. The ranges for Reynolds Number are: 0–175 for smallest, 0–350 for smaller, and 175–350 for small. The ranges for pressure are: 1–1.25 for low, 1–1.5 for medium, and 1.25–1.5 for high. The ranges for curve height are: 5–6 for small, 5–7 for medium, and 6–7 for high. The ranges for % loss are: 0.1–0.55 for small, 0.1–1 for medium, and 0.55–1 for large. The ranges for flow rate are: 0–525 for small, 0–1050 for medium, and 525–1050 for high. The ranges for velocity are: 0–7.5 for small, 0–15 for medium, and 7.5–15 for high. MATLAB simulation was done on the basis of the following equations:

$$R_{e}=\frac{\rho VD}{\mu },\;Q=\frac{\pi R^{4}\Delta P}{8\eta D},\;Q=AV.$$

**Figure 3:**
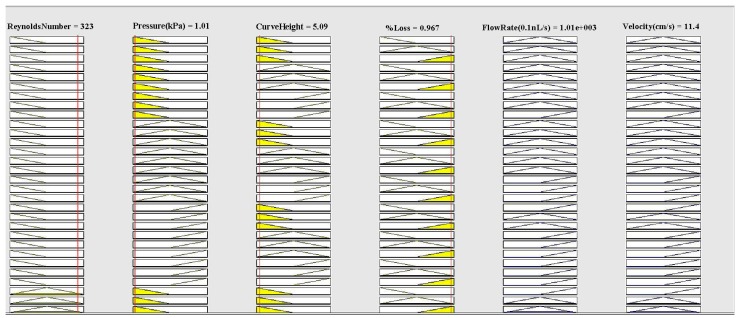
Rule Viewer (MATLAB).

Here Reynolds number = $R_{e}$, velocity = $U_{in}$, viscosity = μ, curve height = $D_{h}$, fluid density = ρ, diameter = *d*, velocity = V, and flow rate = Q. The rule viewer of MATLAB is shown in Figure 3.

**Figure 4:**
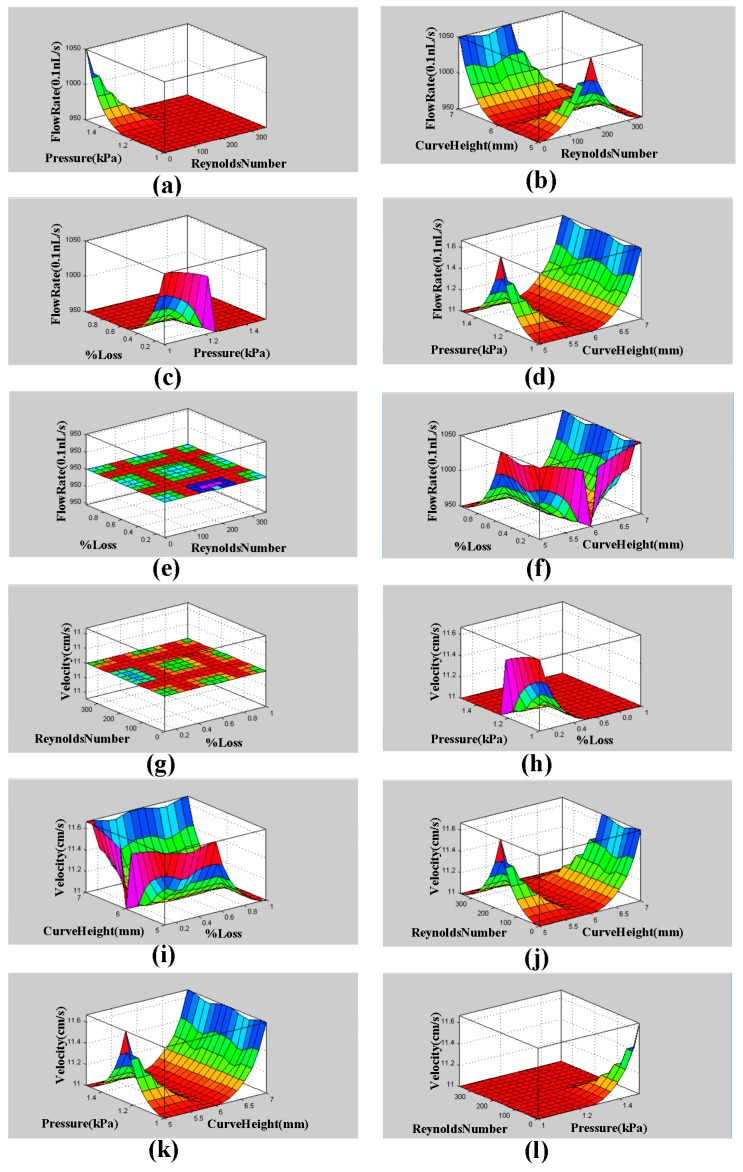
The graphs of surface viewer (3D): (**a**) Flow rate relies upon pressure and Reynolds number; (**b**) Flow rate relies upon Reynolds number and curve height; (**c**) Flow rate relies upon pressure and % loss; (**d**) Flow rate relies upon curve height and pressure; (**e**) Flow rate relies upon Reynolds number and % loss; (**f**) Flow rate relies upon curve height and % loss; (**g**) Velocity relies upon % loss and Reynolds number; (**h**) Velocity relies upon % loss and pressure; (**i**) Velocity relies upon % loss and curve height; (**j**) Velocity relies upon curve height and Reynolds number; (**k**) Velocity relies upon pressure and curve height; (**l**) Velocity relies upon Reynolds number and pressure.

The surface viewer graphs (3D) of flow rate and velocity with pressure, curve radius, and % loss are shown below in Figure 4. The MATLAB function generates these three-dimensional graphs. These graphs show the dependence of one parameter on the two others.

Mamdani’s value, MATLAB simulation results, difference, and error percentage are given in Table 2 below.

The results of this simulation are presented in Table 2. In order to confirm the MATLAB simulation results, Mamdani’s model was used. With the of Mamdani’s relation, the results of velocity and flow rate were 11.5 cm/s and 1001.2 (0.1 nL/s), respectively. MATLAB values for velocity and flow rate measured as 11.4 cm/s and 1001.0 (0.1 nL/s), respectively. The percentage errors for velocity and flow rate were 0.85% and 0.02% respectively. The results are very close. In the succeeding segment, blood rheology is discussed for the justification of the model used for fluid flow before the ANSYS simulation and to confirm the above results for DSMC.

## 4. ANSYS Fluent Simulation for DSMC

This section has been added with the aim of illustrating some of the capabilities of ANSYS Fluent as a powerful simulation tool that gives an accurate analysis of the silver-based descending sinusoidal microchannel. Design Modeler is a designer tool of ANSYS that can create and modify the geometry for its analysis in ANSYS Workbench by using different steps like extrude, sweep, etc. Meshing is the second essential part of the simulation process. Through the optimized meshing, accurate results of flow rate and speed are obtained. Setup, solution, and results optimization in ANSYS are the final parts of the simulation process. The K-epsilon model was carefully chosen from the setup tool of ANSYS, and the power law model was also chosen due to the rheological properties of blood. The estimated calculations of Reynolds and Dean numbers for DSMC were obtained with the help of the equations given below:

Re=ρDhUinμ, De=Red2r.

Here Reynolds number = $R_{e}$, velocity = $U_{in}$, viscosity = μ, hydraulic diameter = $D_{h}$, fluid density = ρ, diameter = *d*, and radius = *r* for DSMC.

**Figure 5:**
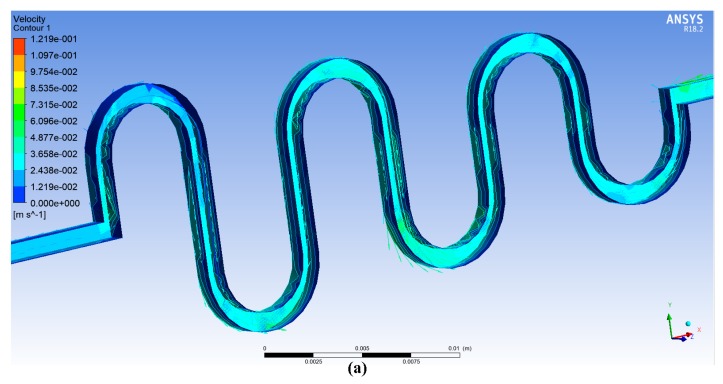

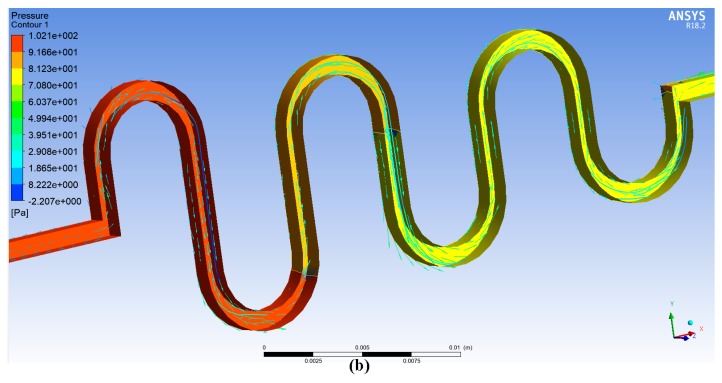
(**a**) Velocity contour plot; (**b**) Pressure contour plot for the descending sinusoidal microchannel (DSMC).

Twenty iterations for the ANSYS simulation were performed with the same conditions of previous study (Afzal et al. 2017: ASMC) [2]. The contours of velocity and pressure are shown in Figure 5a,b.

**Figure 6:**
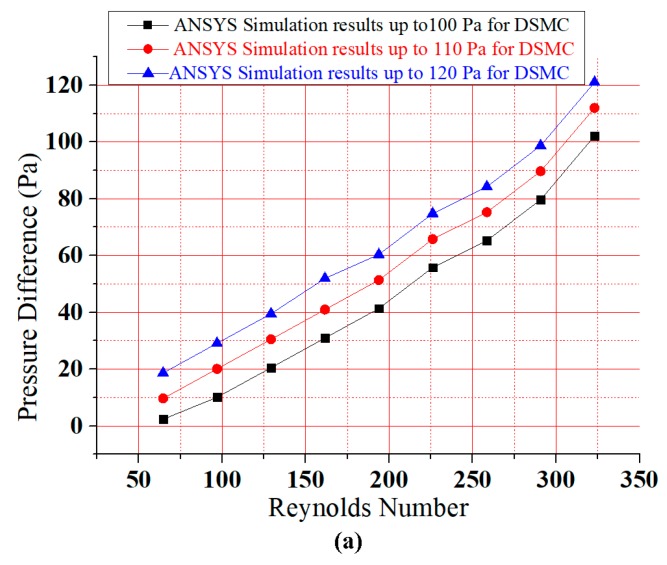

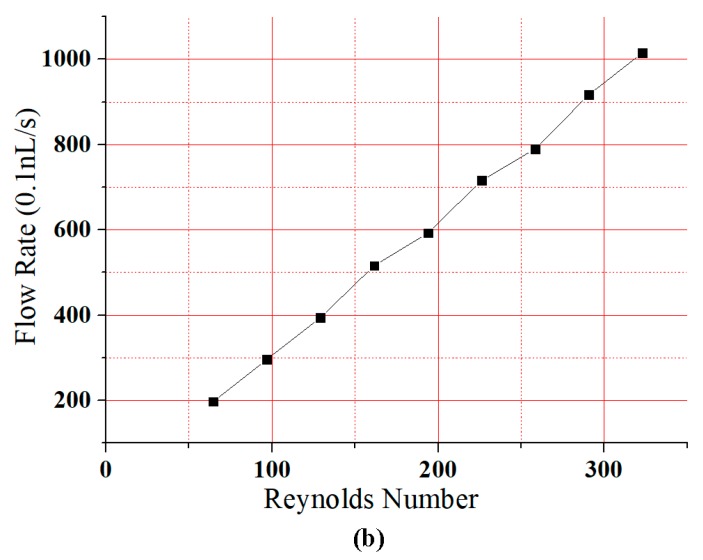
(**a**) The graph between Reynolds number and pressure difference; (**b**) Flow rate vs. Reynolds number for DSMC.

Pressure is increased at the input and reduced at the output of the DSMC. Therefore, a direct relation is present between Reynolds number and pressure difference. However, there are also three descending curves involved in this extension of previous research. Consequently, based on the graphical presentation in Figure 6a, it can be seen that the fluid flows through DSMC gives a better upsurge in pressure difference than ASMC [2]. The simulation through ANSYS was done to detect the flow rate and velocity parameters for DSMC.

Figure 6a shows that the pressure difference increased gradually with Reynolds number. The results for all curves were analogous to the investigations of Chiam et al. (2016) and Afzal et al. (2017) for sinusoidal microchannels (SMCs) and the ASMC, respectively [44]. Due to the dominance of viscid forces, the flow was truly laminar. Therefore, the Reynolds number in this study was taken as just below 350. Figure 6b presents a linear relationship between Reynolds number and flow rate. The rate of flow and velocity were obtained as 1015.3 (0.1 nL/s) and 12.19 cm/s at a Reynolds number of 323. This research shows an agreement between the results of Fuzzy Logic MATLAB and ANSYS simulations. 

## 5. Fabrication of DSMC and Experimental Setup

**Figure 7:**
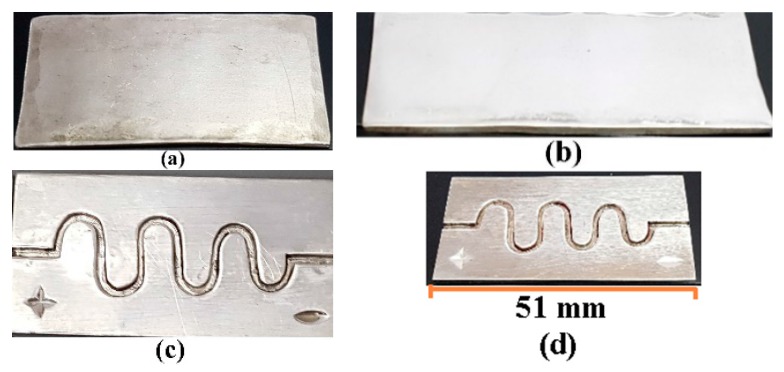
Simple micromachining fabrication of DSMC [2]: (**a**) Raw silver substrate; (**b**) Electro-polishing and ultra-clean surface; (**c**) The real fabrication of the DSMC; and (**d**) Scale of the DSMC.

The channel was fabricated on silver by using a micromachining process [1]. The diameter of the DSMC 900 micron. This process can be used to achieve product dimensions in micron scale. In this fabrication procedure, a callous and sharp knife of the cutting machine AgieCharmilles FORM 1000 (Georg Fischer Ltd, Schaffhausen, Switzerland) was used to eliminate the minor chips from the silver substrate with the relative motion of speed and feed [2,45]. The silver substrate was fabricated with similar dimensions as in our previous study. The fabricated DSMC is shown in Figure 7.

In order to confirm the ANSYS software results, the experimentation was accomplished after the fabrication process. The flow rate and velocity parameters were obtained with the fabricated DSMC. A piezoelectric diaphragm micropump mP6 was employed with the electronic circuit for the experimentation. The clotting of blood was prevented in the micropump with the use of heparin admixed in the blood before use. The mP6 micropump pressurized the blood flow with 55,000 Pa and 0–7 mL/min flow rate. These pressure values can be varied with some adjustments in the mP6. A commercially-available electronic circuit (mP6-EVA, Rise Technology, Santa Clara, CA, USA) was used with mP6, which permitted the adjustment of pumping factors which is done partly with the outer regulation. The source voltages could be given with the help of USB. It was attached to the power supply (USB) and the parameter estimation began. Electric power could be provided by a 2.5–5 V source, and this circuit could produce 270 V; its output power was significantly small. There were connectors and jumpers present in this module. Connector number 1 was the screw terminal which was used for the external power supply. It was also used for the exterior clock or the signal amplitude. Connector number 2 was the solder element for the extension cable and it was used for the connection of the mP6 micropump (Rise Technology, Santa Clara, CA, USA). Connector number 3 was called the Molex, used for the connection of the mP6 pump with USB to provide supply voltage. There were three jumpers present in this circuit: JP1, used for setting the frequency of the micropump; JP2, used to set the amplitude; and JP3, used for setting the supply. There was also a variable resistor for the amplitude adjustment.

**Figure 8:**
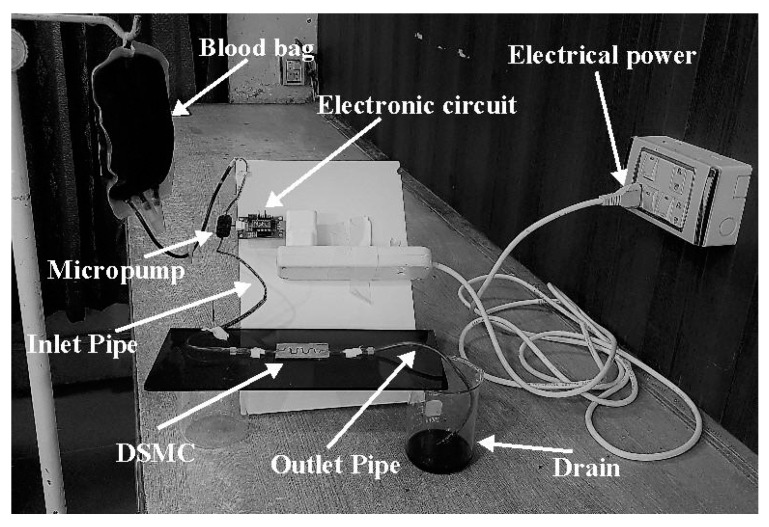
Experimental test setup.

The DSMC was roofed strongly by glass glue and glass pane. In this way, the outflow of blood was prevented. The results were obtained after the experiment, which verified the ANSYS results. The whole experimental apparatus is presented in Figure 8.

The mP6 micropump was utilized with a supplementary electrical circuit (mP6-EVA). The blood had high viscosity and it would certainly clot inside micropump. Consequently, heparin was blended with the blood in order to decrease its viscosity. This technique prevented the blood from clotting inside the micropump. The pump was able to estimate all the required parameters for this study. This module contained jumpers and connectors attached with the controller. Therefore, the required pressure could be adjusted. In order to implant the DSMC (bioengineered vein) in a human body, the line of action and procedural plan should be made. The particular area of the varicose vein would be efficiently shaved and anesthetized. The DSMC would be separated before implantation. The implant would be done by a tiny duct (catheter) made of biocompatible materials. This varicose vein operation is analogous to the angioplasty procedure. The Holter Monitor will observe heart rate and blood pressure. If the doctor is satisfied with procedure, then reasonable blood flow would be reinstated after the operation [2].

## 6. Results and Discussion

At a Reynolds number of 323, 1001.0 (0.1 nL/s) flow rate and 11.4 cm/s velocity were calculated by using Fuzzy logic simulation. At the same Reynolds number, 1015.3 (0.1 nL/s) flow rate and 12.19 cm/s velocity were calculated by using ANSYS software. DSMC was simulated twice with very close and fine results in this study. Fuzzy logic is actually the parametric approximation component of the MATLAB software. An important aspect of this is its ability to show the performance of FLC systems by using mathematical models. The ANSYS software is a dominant computational fluid dynamics (CFD) tool. This software has widespread modeling abilities, and blood flow can be analyzed accurately. The discussion is provided below.

### 6.1. Experimental Results

**Figure 9:**
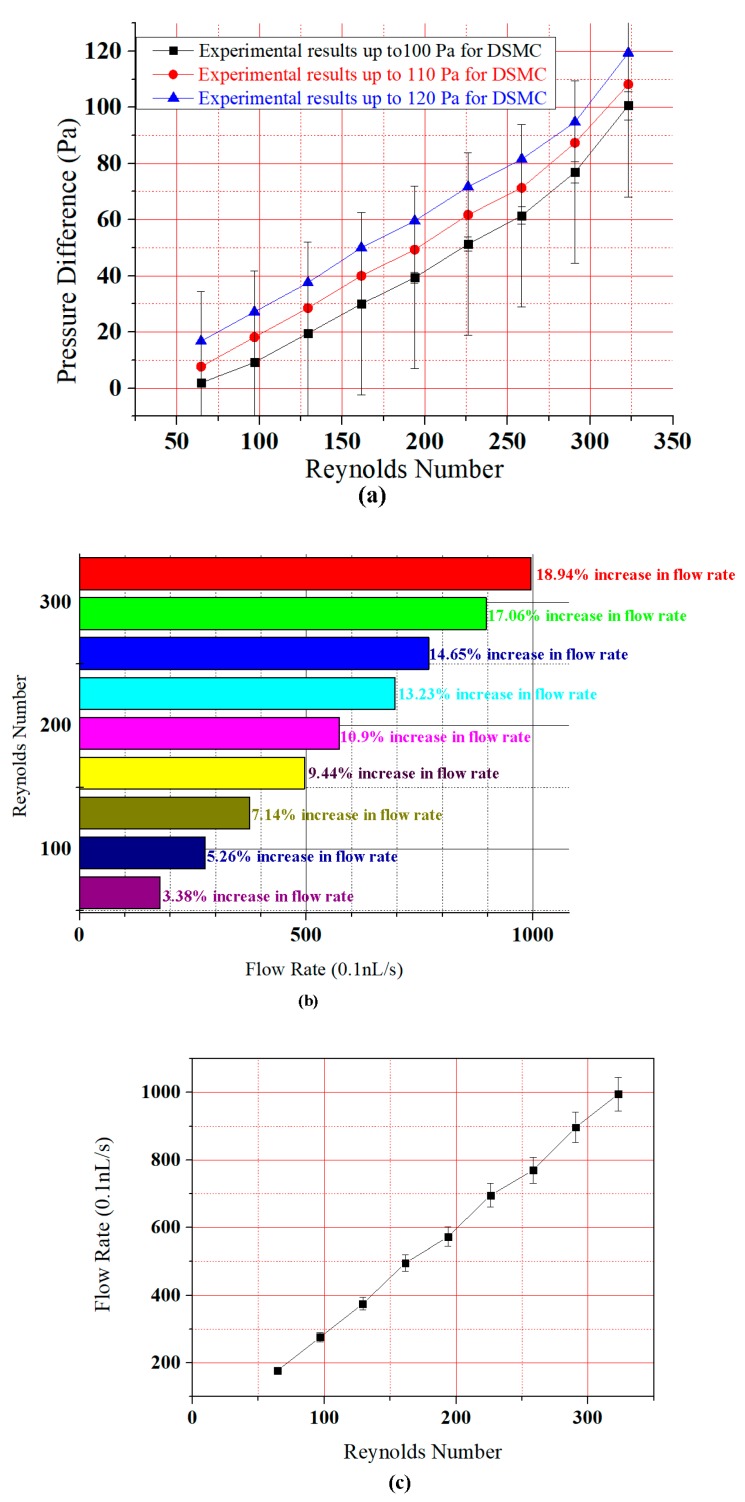
Experimental graphical results (**a**) Graph relating Reynolds number and pressure difference with error bars; (**b**) bar graph between Flow rate and Reynolds number for DSMC; (**c**) graph between Flow rate and Reynolds number with error bar.

The experimental results are shown in Figure 9. In Figure 9a, the pressure difference first increased almost linearly, and then there was an upsurge of pressure—especially beyond the Reynolds number 250. This is because of the descending nature of the DSMC. Blood flow in descending order is always increased. There was also the DSMC which was tested in an experiment for the blood flow. The results in Figure 9 clearly show that there was a sudden upsurge of pressure difference as compared to ASMC [2]. Note the error bars in Figure 9a; the statistical standard deviation calculated from the error bars was 32.47865, and the 5% pressure difference for the curves was 5.03, 3.843, 3.0725, 2.565, 1.9695, 1.4995, 0.977, 0.459, 0.0945 for all points (minimum to maximum) of the graph.

Figure 9b,c shows that the flow rate increased gradually with Reynolds number. There are nine points in the graph which show the percentage increase. At the first point, the simulated increase in flow rate was 3.63% whereas in the experimental result it was 3.38%. At the second point, the simulated increase in flow rate was 5.45%, whereas in the experimental result it was 5.26%. At the third point, the simulated increase in flow rate was 7.27%, whereas in the experimental result it was 7.14%. At the fourth point, the simulated increase in flow rate was 9.49%, whereas the experimental result was 9.44%. At the fifth point, the simulated increase in flow rate was 10.94%, whereas the experimental result was 10.9%. At the sixth point, the simulated increase in flow rate was 13.16%, whereas the experimental result was 13.23%. At the seventh point, the simulated increase in flow rate was 14.54%, whereas the experimental result was 14.65%. At the eighth point, the simulated increase in flow rate was 16.87%, whereas the experimental result was 17.06%. Finally, at the ninth point the simulated increase in flow rate was 18.68%, whereas the experimental result was 18.94%. This data show that there was a small decrease in flow rate in the experiment compared to simulation. However, the results are very close. The standard deviation calculated from the error bars in Figure 9c was 279.58033 for each point, and the 5% pressure difference for the curves was 49.765, 44.825, 38.5, 34.765, 28.625, 24.79, 18.75, 13.815, and 8.875 for all points in the graph. Here, in comparison with previous paper results there is a better flow of blood in the DSMC. Blood flow is the motion of blood through the human body. The deceleration of blood flow is resistance. Blood applies a force on the veins and vessels, which becomes blood pressure. In this study, the pulse pressure was used, which is approximately 120 to 80 Pa in smaller varicose veins [2,46]. 

### 6.2. Dean Number

The Dean number was obtained for each curve of the DSMC [2]. DSMC curves with 7 mm radius of curvature had the Dean numbers 80.75, 74.25, 64.625, 56.5, 48.45, 40.375, 32.3, 24.225, and 16.15. Curves with 6 mm radius of curvature had the Dean numbers 87.21, 80.19, 69.795, 61.02, 52,326, 43.605, 34.884, 26.163, and 17.442. Curves with 5 mm radius of curvature had the Dean numbers 96.9, 89.1, 77.5, 67.8, 58.14, 48.45, 38.76, 29.07, and 19.38. These statistics demonstrate the direct relationship between Reynolds and Dean numbers. The Dean number decreases with the increase of curve radii. Consequently, there were no Dean vortices established, even in the curves of the DSMC [2].

### 6.3. The Role of Diameter and Viscosity of the Vein and Tissue Damage with the Flow of Blood

Blood pressure decreases irregularly when it moves from arteries, capillaries, and veins to much smaller linked vessels [47]. The blood velocity varies as the blood moves from arteries to arterioles to capillaries, and vice versa. In veins, the velocity increases when blood goes back to the heart. The diameter of the blood vessels plays a dramatic role in the variation of flow rate and velocity [47,48]. The thickness of the blood affects its flowing capacity. This is because of erythrocytes (formed elements) and plasma proteins. Generally, the blood viscosity does not change over smaller distances and time periods. When viscosity changes, blood pressure changes [49,50]. The tissue damage presents as the damage of muscles, ligaments, and tendons due to more or less blood flow in smaller veins [2,12]. When the blood flow is normal, then no tissue damage will occur. That is why there is a need for a proper microdevice (DSMC) for implantation.

### 6.4. Implantable Devices in Humans

The definition of the word “implant” is the insertion of an organ or device in the human body in a surgical process. The history of metal implants is very old, dating from 1875 to the present for pure silver, gold, and copper. Polymers and ceramics are now also used for implantation. Intraocular lenses, cochlear implants, brain implants, heart implants (pacemakers, heart valves, stents), joint implants, wearable artificial kidneys (WAKs), and artificial dermis are implantable devices for humans.

### 6.5. Comparison of ASMC and DSMC 

In first part of this study, we discussed our previous work (Afzal et al., 2017), wherein we developed the ASMC as an implant for varicose veins [2]. It was found that the flow rate and velocity was low because of the ascending nature of the ASMC. Now in this second phase of the study, the authors have found that the flow rate and velocity was much better due to the descending nature of the DSMC. Simulations were done with the same boundary conditions. Fabrication was done with the same process, and the same experimental technique was used in this study as well. All of the surgical problems with their severe effects were discussed previously [2]. The authors came to know that the ASMC cannot be implanted at those places where increased flow rate and velocity are required [35,51]. Therefore, the DSMC was simulated twice, fabricated, and tested experimentally to evaluate the possibility of implantation in human varicose veins. As a fluid, blood has high viscosity, and the flow was not complex in this microchannel. Therefore, neither channel showed Dean vortices, especially in the curved parts of the microchannel. The flow displayed a small Reynolds number. Therefore, the Dean numbers were also relatively low. Dean vortices are prominent in larger channels with less-viscous fluid and complex flow. This microchannel has straight parts between the curves and blood flowed steadily through them. However, the Dean number was calculated and found to be even lower than the Reynolds number.

### 6.6. Results Comparison

The three major steps of this research show a clear agreement between simulation and experimental results. The MATLAB results were 1005.8 (0.1 nL/s) flow rate and 11.8 cm/s velocity. The ANSYS results were 1015.3 (0.1 nL/s) and 12.19 cm/s velocity. Finally, the experimental results showed 995.3 (0.1 nL/s) flow rate and 12.2 cm/s velocity. All of the results were at the same Reynolds number (323). The results are shown in Table 3 below.

Although there is no data for the DSMC with flow rate and velocity parameters for verification, these parameters have been measured with other channels [2,52]. The deviance in results can be explained by many factors, namely friction, pressure, and the ascending and descending nature of the microchannel. These features can modify the results. The authors have performed the current study with real and ideal conditions by changing the parameters. Fuzzy conditions were very close to ideal conditions and were found to be more accurate than ANSYS, because it provides thousands of infinite values between 0 and 1, and is therefore more reliable. Fuzzy controllers have been used in many appliances, such as blood pressure apparatuses, glucometers, washing machines, dryers, air conditioners, etc. ANSYS has a different environment than MATLAB Fuzzy logic. After the geometry was created in the design modeler, the meshing technique was used. In meshing, the mesh size is very important. Sometimes simulation is not done for every mesh size. Therefore, a proper mesh size must be created for the correct simulation. For the ANSYS results, the authors have taken twenty iterations for each simulation process. The testing was very close to the ideal and real environments. Therefore, the Fuzzy results were very close to the testing results. The testing was done many times at different ideal values of pressure.

## 7. Conclusions

The objective of this work was to conduct MATLAB and ANSYS simulations, fabrication, and experimentation with the DSMC. Moreover, the DSMC (bioengineered vein) was used to determine the velocity and the flow rate of blood. The flow of blood was without Dean vortices. Additionally, a complete evaluation was performed comparing experiment and simulation. Silver was used because of its cheapness and biocompatibility. These bioengineered veins can be inserted in place of varicose veins for the sufficient flow of blood. This microdevice (DSMC) can be implanted at those places where the ASMC cannot be implanted. For this objective, the DSMC must be parted from the silver substrate for implant as the bioengineered vein. DSMCs of various designs and dimensions can also be prepared as required. The present study has some limitations. The DSMC was restricted for single-phase streamline flow. In this study, valves were neglected and the natural elasticity was present in the veins because of the very small size of the DSMC. The final restriction in this study was the silver material. We will use polymer materials for the fabrication of ASMCs and DSMCs in the future.

## Figures and Tables

**Table 1:** Medical devices implant in human body. ASMC: ascending sinusoidal microchannel.

References	Medical Device	Organ	Material/Type	Software
Afzal et al. [2]	ASMC	Varicose vein	Silver	ANSYS, MATLAB
Sundell et al. [26]	Dental implant	Human jaw	Titanium	IVAS 3.6.6 software
Küçük et al. [27]	Cardiovascular implant	Heart	Electronics	Corrosion Analyzer
Dai et al. [28]	Intraocular lenses	Eyes	Plastic	ANSYS
Todd and Naghdy [29]	Cochlear implant	Ears	Electronics	ANSYS
Zhang et al. [30]	Intracranial electroencephalogram electrodes	Brain	Electronics	Finite element simulation
Georgia et al. [31]	Heart stent	Heart	Metal or plastic	ANSYS
Shah et al. [32]	Knee implant	Knee	Titanium and Stainless Steel	ANSYS
Kim et al. [33]	Wearable artificial kidney (WAK)	Kidney	Charcoal, activated carbon, and zeolite	ANSYS Fluent
Zak et al. [34]	Artificial dermis	Skin	Donated skin tissues and polymers	ANSYS

**Table 2:** Simulated results with Mamdani’s results.

Model	Flow Rate in 0.1 nL/s	Velocity in cm/s
Mamdani’s value	1001.2	11.5
MATLAB simulation	1001.0	11.4
Difference	0.2	0.1
Error percentage	0.02%	0.85%

**Table 3:** The results of simulations and experiment.

MATLAB Results	ANSYS Results	Experimental Results
Reynolds Number = 323	Reynolds Number = 323	Reynolds Number = 323
Flow Rate = 1001.0 (0.1 nL/s)	Flow Rate = 1015.3 (0.1 nL/s)	Flow Rate = 995.3 (0.1 nL/s)
Velocity = 11.4 cm/s	Velocity = 12.19 cm/s	Velocity = 12.2 cm/s

## References

[1] Anwar M.A., Adesina-Georgiadis K.N., Spagou K., Vorkas P.A., Li J.V., Shalhoub J., Holmes E., Davies A.H. (2017). A comprehensive characterisation of the metabolic profile of varicose veins; implications in elaborating plausible cellular pathways for disease pathogenesis. Sci. Rep..

[2] Afzal M.J., Tayyaba S., Ashraf M.W., Hossain M.K., Uddin M.J., Afzulpurkar N. (2017). Simulation, fabrication and analysis of silver based ascending sinusoidal microchannel (ASMC) for implant of varicose veins. Micromachines.

[3] Hirai M., Naiki K., Nakayama R. (1990). Prevalence and risk factors of varicose veins in Japanese women. Angiology.

[4] Sansilvestri-Morel P., Rupin A., Badier-Commander C., Kern P., Fabiani J., Verbeuren T.J., Vanhoutte P.M. (2001). Imbalance in the synthesis of collagen type I and collagen type III in smooth muscle cells derived from human varicose veins. J. Vasc. Res..

[5] Tessari L., Cavezzi A., Frullini A. (2001). Preliminary experience with a new sclerosing foam in the treatment of varicose veins. Dermatol. Surg..

[6] Blomgren L., Johansson G., Dahlberg-Åkerman A., Norén A., Brundin C., Nordström E., Bergqvist D. (2004). Recurrent varicose veins: Incidence, risk factors and groin anatomy. Eur. J. Vasc. Endovasc. Surg..

[7] Lawaetz M., Serup J., Lawaetz B., Bjoern L., Blemings A., Eklof B., Rasmussen L. (2017). Comparison of endovenous radiofrequency ablation, laser ablation, foam sclerotherapy and surgical stripping for great saphenous varicose veins. Extended 5-year follow-up of a RCT. Int. Angiol..

[8] Belcaro G., Dugall M., Luzzi R., Corsi M., Ledda A., Ricci A., Pellegrini L., Cesarone M.R., Hosoi M., Errichi B.M. (2017). Management of varicose veins and chronic venous insufficiency in a comparative registry with nine venoactive products in comparison with stockings. Int. J. Angiol..

[9] Dabbs E., Nemchand J.L., Whiteley M.S. (2017). Suprapubic varicose vein formation during pregnancy following pre-pregnancy pelvic vein embolisation with coils, without any residual pelvic venous reflux or obstruction. SAGE Open Med. Case Rep..

[10] Bothra N., Panda L., Sheth J., Tripathy D. (2017). Role of intralesional bleomycin sclerotherapy as the sole or adjunct treatment of superficial ocular adnexal lymphatic malformations. Eye.

[11] Saleh M.S. (2017). Duplex ultrasound guided catheter directed foam sclerotherapy for treatment of axial varicose veins of the lower limbs and its preliminary results. Int. J. Surg. Res..

[12] Pires M.F.B., Nogueira R.F., Navarro T.P. (2017). Chronic Venous Disease and Varicose Veins, in Vascular Diseases for the Non-Specialist.

[13] Rao S.V.S., Datta A.S., Anvesh D. (2017). Spectrum of chronic venous insufficiency of lower limbs-A katuri perspective. J. Evid. Based Med. Healthc..

[14] Argyriou C., Papasideris C., Antoniou G.A., Georgakarakos E., Papanas N., Lazarides M.K., Georgiadis G.S. (2017). The effectiveness of various interventions versus standard stripping in patients with varicose veins in terms of quality of life. Phlebology.

[15] Hager E.S., Ozvath K.J., Dillavou E.D. (2017). Evidence summary of combined saphenous ablation and treatment of varicosities versus staged phlebectomy. J. Vasc. Surg. Venous Lymphat. Disord..

[16] Zafarghandi M.R., Farsavian H., Davoudi M., Farsavian A.A. (2017). Outcome of ultrasonography-guided foam sclerotherapy versus stab avulsion ambulatory phlebectomy in the treatment of varicosis in small veins of the leg: A single blinded randomized clinical trial. Iran. J. Radiol..

[17] Rajam V., Kathaperumal K.D.A., Hemant U., Rajavelu N. (2017). Study of clinical outcomes of subfascial perforator ligation surgery in perforator incompetence. J. Evid. Based Med. Healthc..

[18] Lin Y.-N., Hsieh T.-Y., Huang S.-H., Liu C.-M., Chang K.-P., Lin S.-D. (2017). Management of venous ulcers according to their anatomical relationship with varicose veins. Phlebology.

[19] Kumar S.P., Ramesh R., Aravind T. (2017). Analysis of different size microchannel through particle tracing for biomolecule separation. J. Comput. Theor. Nanosci..

[20] Huang L., Zhao P., Bian S., Shi G., Liu P., Zong S., Wang W. A novel bioMEMS device for efficient on-chip single cell loading and 3D rotation. Proceedings of the 2017 IEEE 30th International Conference on Micro Electro Mechanical Systems (MEMS).

[21] Wang S.-L., Fan S.-K. DNA stretching in surfactant-stabilized microchannel. Proceedings of the 2017 19th International Conference on Solid-State Sensors, Actuators and Microsystems (TRANSDUCERS).

[22] Faustino V., Catarino S.O., Lima R., Minas G. (2016). Biomedical microfluidic devices by using low-cost fabrication techniques: A review. J. Biomech..

[23] Hu W., Ohta A.T. (2017). Editorial for the Special Issue on Microdevices and Microsystems for Cell Manipulation.

[24] Birch C., Landers J.P. (2017). Electrode materials in microfluidic systems for the processing and separation of DNA: A mini review. Micromachines.

[25] Pinto E., Faustino V., Rodrigues R.O., Pinho D., Garcia V., Miranda J.M., Lima R. (2014). A rapid and low-cost nonlithographic method to fabricate biomedical microdevices for blood flow analysis. Micromachines.

[26] Sundell G., Dahlin C., Andersson M., Thuvander M. (2017). The bone-implant interface of dental implants in humans on the atomic scale. Acta Biomater..

[27] Küçük M., Çömlekoğlu M.E., Zor M. (2010). The effect of crown geometry on stress distribution of a single implant restoration: A finite element analysis. Turkiye Klinikleri J. Dent. Sci..

[28] Dai P., Wang B., Bao C., Ju Y. (2010). Constructing a computer model of the human eye based on tissue slice images. J. Biomed. Imaging.

[29] Todd C., Naghdy F. Virtual cochlear implant insertion for medical education. Proceedings of the Eurohaptics Conference, 2005 and Symposium on Haptic Interfaces for Virtual Environment and Teleoperator Systems.

[30] Zhang W., Li Z., Gilles M., Wu D. (2014). Mechanical simulation of neural electrode-brain tissue interface under various micromotion conditions. J. Med. Biol. Eng..

[31] Karanasiou G.S., Sakellarios A.I., Tripoliti E.E., Petrakis E.G.M., Zervakis M.E., Migliavacca F., Dubini G., Dordoni E., Michalis L.K., Fotiadis D.I. Modeling stent deployment in realistic arterial segment geometries: The effect of the plaque composition. Proceedings of the 2013 IEEE 13th International Conference on Bioinformatics and Bioengineering (BIBE).

[32] Shah Y., Bhave A., Sonetha V. (2015). Fatigue analysis of the knee joint. Procedia Comput. Sci..

[33] Kim S., Feinberg B., Kant R., Chui B., Goldman K., Park J., Moses W., Blaha C., Iqbal Z., Chow C. (2016). Diffusive silicon nanopore membranes for hemodialysis applications. PLoS ONE.

[34] Zak J., Hadas Z., Dusek D., Pekarek J., Svatos V., Janak L., Prasek J., Hubalek J. (2016). The charge push-through electronics design for fully implantable artificial cochlea powered by energy harvesting technologies. Microsyst. Technol..

[35] Baskurt O.K., Meiselman H.J. (2003). Blood rheology and hemodynamics. Seminars in Thrombosis and Hemostasis.

[36] Skalak R., Keller S., Secomb T. (1981). Mechanics of blood flow. J. Biomech. Eng..

[37] Zhang J., Zhang P., Fraser K.H., Griffith B.P., Wu Z.J. (2013). Comparison of fluid dynamic numerical models for a clinical ventricular assist device and experimental validation. Artif. Organs.

[38] Shih T.-C., Hsiao H.-D., Chen P.-Y., Tu C.-Y., Tseng T.-I., Ho Y.-J. (2014). Study of pre-and post-stent implantation in the trachea using computational fluid dynamics. J. Med. Biol. Eng..

[39] Qian Y., Liu J.L., Itatani K., Miyaji K., Umezu M. (2010). Computational hemodynamic analysis in congenital heart disease: Simulation of the Norwood procedure. Ann. Biomed. Eng..

[40] Heck M.L., Yen A., Snyder T.A., O'Rear E.A., Papavassiliou D.V. (2017). Flow-field simulations and hemolysis estimates for the food and drug administration critical path initiative centrifugal blood pump. Artif. Organs.

[41] Rahman I.A. (2017). Computational fluid dynamics as a tool for testing functional and ecological hypotheses in fossil taxa. Palaeontology.

[42] Cherng W.-J., Dong Z.-S., Chou C.-C., Yeh C.-H., Pan Y.-H. (2017). Hydrodynamic simulation of an orbital shaking test for the degradation assessment of blood-contact biomedical coatings. Micromachines.

[43] Elblbesy M.A., Hereba A.T. (2016). Computation of the coefficients of the power law model for whole blood and their correlation with blood parameters. Appl. Phys. Res..

[44] Chiam Z.L., Lee P.S., Singh P.K., Mou N. (2016). Investigation of fluid flow and heat transfer in wavy micro-channels with alternating secondary branches. Int. J. Heat Mass Transf..

[45] Folch A. (2016). Introduction to bioMEMS.

[46] Nicolaides A.N., Zukowski A.J. (1986). The value of dynamic venous pressure measurements. World J. Surg..

[47] Fung Y.-C. (2013). Biomechanics: Circulation.

[48] Smieško V., Kožík J., Doležel S. (1985). Role of endothelium in the control of arterial diameter by blood flow. J. Vasc. Res..

[49] Letcher R.L., Chien S., Pickering T.G., Sealey J.E., Laragh J.H. (1981). Direct relationship between blood pressure and blood viscosity in normal and hypertensive subjects: Role of fibrinogen and concentration. Am. J. Med..

[50] Fu G.-X., J M., Han L.-Z., Xu C.-Z., Pan F.-F., Hu T.-J., Zhong Y. (2017). Erythrocyte rheological properties but not whole blood and plasma viscosity are associated with severity of hypertension in older people. Zeitschrift für Gerontologie und Geriatrie.

[51] Anand M., Rajagopal K.R. (2017). A short review of advances in the modelling of blood rheology and clot formation. Fluids.

[52] Bento D., Sousa L., Yaginuma T., Garcia V., Lima R., Miranda J.M. (2017). Microbubble moving in blood flow in microchannels: Effect on the cell-free layer and cell local concentration. Biomed. Microdevices.

